# The Role of the Gut Microbiota in the Development of Ischemic Stroke

**DOI:** 10.3389/fimmu.2022.845243

**Published:** 2022-03-28

**Authors:** Jinchen Wang, Hongfei Zhang, Jianying He, Xiaoxing Xiong

**Affiliations:** ^1^ Department of Anesthesiology, Zhujiang Hospital of Southern Medical University, Guangzhou, China; ^2^ Central Laboratory, Renmin Hospital of Wuhan University, Wuhan, China; ^3^ Department of Orthopedic, JiangXi Provinvcial People’s Hospital, The First Affiliated Hospital of Nanchang Medical College, Nanchang, China

**Keywords:** ischemic stroke, gut microbiota, gut-brain axis communication, neuro inflammation, fecal microbiota transplantation

## Abstract

An increasing number of studies have focused on the gut microbiota and its relationship with various neurological diseases. The gut microbiota can affect the metabolic status of the body, in addition to having an important impact on blood pressure, blood glucose, and atherosclerosis, all of which are risk factors for ischemic stroke. In this review, we summarized studies that included the physiological function of the gut microbiota and gut microbiota disorders related to the central nervous system, thus providing novel ideas for the prevention and treatment of ischemic stroke.

## Introduction

Ischemic stroke is a common central nervous system (CNS) disease and one of the most serious health problems worldwide. The gut microbiota is an important intestinal microecosystem that plays an important role in the homeostasis of the internal environment. Under normal circumstances, intestinal microorganisms and the host maintain a dynamic ecological balance, and gut microbiota homeostasis is closely associated with the health. In recent years, studies have found that the gut microbiota plays a key role in the occurrence and development of ischemic stroke (1–3). An imbalance within the gut microbiota is not only closely related to gastrointestinal diseases, such as irritable bowel syndrome, ulcerative colitis, colon cancer (4–6), but is also associated with the occurrence and development of hypertension, diabetes, obesity, atherosclerosis and metabolic diseases (7–9), all of which are risk factors for ischemic stroke. In this review, we summarized the influence and mechanisms of gut microbiota disorders on the pathogenesis of chronic metabolic diseases and stroke, and provided new proposals for the prevention or treatment of stroke.

## What Is the Human Gut Microbiota Imbalance?

The human gut microbiota is a very complex ecosystem with a large number and wide variety of species involved, which consists of approximately 10 times the number of cells in the human body and > 100 times as many genes as the entire human genome (10). The human gut microbiota is mainly composed of bacteria from the phyla Firmicutes and Bacteroidetes with the rest phyla comprising Actinobacteria, Proteobacteria and Verrucomicrobia (11). The gut microbiota is vital to health and plays a key role in many important physiological functions: (1) physiological nutrition: some probiotics can decompose dietary fiber and starch that cannot be digested in the daily diet and can degrade polysaccharides (12); (2) antagonism: some probiotics reproduce in the intestinal tract and generate various metabolites, such as hydrogen peroxide, carbon dioxide, and acetaldehyde, all of which inhibit or kill intestinal *Escherichia coli* and *Streptococcus* spp. (13); (3) immune regulation: probiotics, such as *Bifidobacterium* spp., can improve the body’s immune response and expression level of multiple cytokines, thus improving the disease-resistance of the host (14); (4) anti-cancer: *Lactobacillus* and *Bifidobacterium* spp. and other probiotics can enhance intestinal peristalsis, which may reduce the intestinal retention time of harmful or carcinogenic substances (15); (5) other functions: normal gut microbiota can regulate the development of the brain (15, 16).

Normally, the gut microbiota exists in a dynamically balanced ecological environment. The gut microbiota imbalance may occur for several reasons, such as prolonged drug treatments, stress, or infections by pathogenic bacteria and viruses. Under these conditions, the number, species, proportion, location, and biological characteristics of the gut microbiota will be altered (17).

## Maturation of the Human Gut Microbiota

The colonization of human gut microbiota in the infant is related to many factors including: intrauterine contact, delivery method (vaginal or caesarean delivery), feeding method (breast feeding or formula milk), the mother’s diet, external environment, and the use of antibiotics. These factors have a significant impact on the composition of the neonatal gut microbiota (18–20). The controversy over whether colonization of the human gut microbiota begins at birth or in the womb resulted from the discovery of the presence of bacteria in the placenta and amniotic fluid by Jimenez et al. (19). Prior to this, the fetus was considered to be a completely sterile environment. In a study of pregnant women who had caesarean deliveries, Collado et al. found that the characteristics of the microbiota in the placenta and amniotic fluid were consistent with the meconium of the newborn (21). The delivery mode can also affect the colonization of the neonatal gut. During vaginal delivery, the neonate is exposed to microorganisms that mainly come from the mother’s vagina and feces, while the composition of the baby’s intestinal flora is similar to that of the mother’s skin after caesarean delivery. Lee et al. found that on day 1 and 3 after birth, the richness and diversity of the gut microbiota of newborns delivered vaginally was significantly higher than that of newborns delivered by caesarean section, but the differences gradually decreased over time (22).

The intestinal microbiota undergoes a series of maturation processes in infancy and early childhood; however, by 3 years of age, it has established a structure similar to that of the adult intestinal microbiota (23). During the first 3 years of age, there is substantial synaptogenesis and neural development occurring in the brain (24). In addition, fewer types of gut microbes are present in early childhood and adolescence. The gut microbiota eventually reaches a relative maturity in adulthood and is manifested by an increase in the numbers and types of gut microbiota that display stronger resistance to antibiotics and other disturbances (25). Interactions between the host and gut microbiota have been shown to occur during an important window of brain development, referred to as “early life” (26). Disruption of the gut microbiota during this period may interfere with communication between the intestinal microbiota and brain, which may lead to CNS diseases (27, 28).

## Experimental Methods to Study the Gut Microbiota and Gut-Brain Axis

The traditional methods used to study the gut microbiota mainly include bacterial cultures and molecular biological techniques that are independent of culturing (29). The former method is mainly used for fecal culture; however, under laboratory conditions, 99% of the microorganisms in feces cannot be isolated and cultured, and these procedures are complicated and have low purification efficiency. These methodologies are laborious, and the bacterial strains obtained are limited. In recent years, real time quantitative PCR, fluorescence *in situ* hybridization, denatured gradient gel electrophoresis, terminal restriction fragment length polymorphism, second generation of sequencing, metagenomics, metabolomics, 16S rRNA sequencing, and other sequencing technologies have been developed (10, 30, 31). Development of germ-free (GF) animal models is an important method to study gut microbiota. GF mice can be colonized with a specific bacterial strain or transplanted with feces (32, 33). Additionally, antibiotics or probiotics can be used. The former interferes with the composition of the normal gut microbiota in mice, while the latter can be used to treat the mice with brain dysfunction. Using these methods, the differences in various physiological indicators and behaviors of mice after different interventions can be analyzed (34).

## Mechanistic of Interactions Between the Gut Microbiota and Brain

### Neural Pathways

The gastrointestinal nervous system is regulated by the CNS, enteric nervous system (ENS), and autonomic nervous system and includes four stages of neural regulation (35). The first stage is the self-regulation of the ENS. The ENS is composed of an intermuscular and submucosal plexus. The sensory and motor neurons of the ENS are connected with each other to process and integrate independent information. The second stage involves the prevertebral ganglion, which accepts information from the ENS and CNS. The third stage involves the CNS, which senses the changes in the environment inside and outside of the intestinal tract. The CNS integrates the information from the brain and spinal cord centers and transmits the signal to the ENS or acts directly on the gastrointestinal effector cells *via* the autonomic nervous system and neuroendocrine system to regulate the activity of smooth muscle, glands, and blood vessels. The last stage consists of developed brain areas, and information from the cortex and subcortical areas interests at specific brainstem nuclei in the basal ganglia. These neural networks connect the CNS and gastrointestinal tract and represent the structural foundation for interactions between the gut microbiota and CNS (36). A disorder at any level of neural control will affect the transmission of information between the brain and intestinal tract.

The ENS can directly transfer information sensed in the gut to the brain *via* the enteric and vagal afferent nerves. The vagus nerve pathway is the main pathway by which the gut microbiota affects the CNS. Studies have shown that gut microbes activate the vagus nerve, which plays a key role in brain function, metabolism, and many behavioral changes in animal models (37–39).

### Neurotransmitter Endocrine Pathways

Biochemical changes in the brain *via* the hypothalamic-pituitary-adrenal (HPA) axis can lead to changes in intestinal physiology. The HPA axis activates a stress response that affects intestinal permeability, motility, and mucus production, thereby changing the intestinal environment and affecting the composition and activity of the intestinal microbiota. Sudo et al. showed that the concentrations of corticosteroids and adrenal hormones in GF mice were higher than normal mice under stress conditions, which demonstrated that the gut microbiota was associated with the HPA axis (40). Intestinal colonization by *Bifidobacterium* spp. only attenuated the HPA axis response early in life, suggesting that exposure to primitive microbes is necessary to inhibit HPA axis regulation (41).

The gut microbiota can cause changes in behavior and cognitive function by influencing the release of diversified neurotransmitters, including norepinephrine, dopamine, 5-hydroxytryptophan, and brain-derived neurotrophic factor (42). The gut microbiota secretes various neurotransmitters, such as γ-aminobutyric acid, catecholamine and histamine, and transmits signals to the CNS *via* intestinal nerves and enterochromaffin cells (43). It has been found that GF mice show less depressive and anxious behaviors, the striatum contains higher 5-hydroxytryptophan content, and the number of enterochromaffin cells is significantly larger compared with that in specific pathogen-free mice (44). Desbonnet et al. (45) demonstrated that, after *Bifidobacterium* uptake in Sprague-Dawley rats, concentrations of the pro-inflammatory factors like IFN-γ, TNF-α, and IL-6 decreased significantly, but the concentrations of serum tryptophan, 5-hydroxyindoleacetic acid in the frontal cortex, and dopamine in the amygdala increased significantly. These findings suggest that the gut microbiota can influence brain function through complex neurotransmitter pathways, however, confirmation will require additional clinical studies.

### Bacterial Metabolites and Host Metabolic Pathways

Microbes can influence neurophysiological changes in their hosts by metabolizing chemicals that bind to receptors inside and outside of the intestine. Short-chain fatty acids (SCFAs) are produced by microbe-mediated fermentation of dietary fiber and mainly include acetate, propionate, and butyrate (46). SCFAs affect the CNS *via* the following three pathways: (1) binding to the G-protein coupled receptors GPR41 and GPR43 and acting as signaling molecules to activate special immune cells. The process is involved in response to an acute infection or in an abnormality in the regulating mechanism; therefore, leading to increased intestinal permeability and absorption of active metabolites (47); (2) direct activation of the sympathetic nervous system by the GPR41 located on sympathetic neurons (48); and (3) crossing the blood-brain barrier, which affects both nerve signals and neurotransmitter products, thereby leading to behavioral changes (48, 49). Intraperitoneal injection of sodium butyrate improved the breakdown of the blood-brain barrier (BBB) in a rat ischemic stroke model with transient middle cerebral artery occlusion (50). Studies have shown that intravenous or peritoneal administration of sodium butyrate inhibited histone deacetylation (51), prevented BBB decomposition (52), and promoted angiogenesis and neurogenesis (53, 54).

Intestinal bacteria can metabolize tryptophan into active products. For example, *E. coli* uses tryptophan to synthesize indoles, which can reduce the virulence and biofilm formation of *E. coli* and other bacteria in addition to having salutary effects on the intestinal environment (55). Tryptophan metabolites also regulate astrocyte activation and CNS inflammation by activating aromatic hydrocarbon receptors (56).

Dietary phosphatidylcholine, L-carnitine and choline are metabolized by the gut microbiota to produce trimethylamine, which is then oxidized by hepatic flavin monooxygenase to produce trimethylamine N-oxide (TMAO) (57). TMAO can inhibit the reverse transport of cholesterol, affect lipid metabolism in the intestinal tract and liver, activate macrophages, promote the accumulation of foam cells in the blood vessel walls, and increase the risk of atherosclerosis (58). In the past, it was thought that the interactions between gut bacteria and the host mainly took place in the intestine (59); however, recent studies have shown that small amounts of bacteria entering the bloodstream from the gut can cause chronic inflammation throughout the body (60, 61). Systemic chronic inflammation occurs in many chronic metabolic diseases, such as obesity, type 2 diabetes mellitus, and atherosclerosis (62).

## Immunological Mechanisms

The immune system, including both adaptive and innate immunity, plays a crucial role in the gut-brain axis. The gut microbiota can affect the maturation, development, and function of immune cells in the CNS, as well as the activation of peripheral immune cells, involved in both cellular and humoral immunity (63). The gut microbiota affects neurophysiological functions, which includes neural development, CNS immune activation, and BBB integrity, by regulating microglia and astrocyte development and function (64). The gut microbiome also regulates peripheral immune responses and plays a key role in brain inflammation, injury, and behavior (65).

Microglia are the most numerous and abundant immune cells in the brain and account for approximately 5%–20% of all glial cells (66). The immune functions of microglia in the CNS include phagocytosis, antigen presentation, cytokine production, and activation of inflammatory responses (67, 68). The gut microbiota can affect the maturation and function of microglia. Compared with those in normal mice, the numbers of immature microglia in the cortex, hippocampus, olfactory bulb, corpus callosum, and cerebellar gray matter and cerebral white matter in the GF mice were significantly increased (69). Moreover, when compared with normal mice and GF mice, brain microglia showed expanded maturity and increased expression of colony stimulating factor 1 receptor, F4/80, and CD31 in normal mice. The expression of these molecules is present in mature microglia and gradually decrease, and the same phenomenon occurred in normal mice after antibiotic treatment (69). The immune response induced by lipopolysaccharide or choriomeningitis virus included decreased activation of brain microglia, delayed development of immune cells, and reduced production of the inflammatory cytokines IL-6, IL-1β and TNF-α in GF mice compared with normal mice (68).

Astrocytes are the most numerous and largest glial cells in the brain and perform various functions, including regulating BBB integrity, neurotransmitter conversion, ion gradient balance, cerebral blood flow adjustment, and nutrient metabolism (70). Astrocytes integrate information from adjacent glial cells, neurons, blood vessels, and immune cells to regulate neural cells excitability and synaptic formation (71). The intestinal microbiota metabolizes tryptophan, including indole-3-aldehydes and indole-3-propionic acid (72), which in turn, activates the astrocyte aromatic hydrocarbon receptors and regulates astrocyte activity (56).

The intestinal microbiota and mucosal cells regulate the activation of immune molecules that affect the CNS, including the pro-inflammatory factors IL-8 and IL-1 and anti-inflammatory factors IL-10 and TGF-β (73–75). Intestinal mucosal pattern recognition receptors include Toll-like receptors that bind to lipopolysaccharide and other microbial-related molecules and activate immune cells, such as dendritic cells, neutrophils, and macrophages (76). Receptor binding results in the production of pro-inflammatory cytokines that include IL-1α, IL-1β, TNF-α, and IL-6, which in turn can cross the BBB and affect brain function directly (77). One study found that filamentous bacteria promoted the development of T-helper cells that produced IL-17 (Th17) in the small intestine of mice, and further study found that mice lacking normal intestinal flora had very few Th17 cells (78, 79). Lee et al. (80) demonstrated that the lack of normal number of Th17 cells in GF mice resulted in an immune deficiency against pathogenic infections, but the mice had increased resistance to the development of autoimmune diseases, such as autoimmune encephalomyelitis. A study on multiple sclerosis showed that IgA^+^ B cells from the intestine crossed the BBB, migrated to brain lesions, and then released IgA antibodies (81). These antibodies mainly reacted with diversified bacteria mainly, including MS related bacteria, without attacking self-brain tissue, which may play a protective role in ameliorating neuroinflammation (81). In conclusion, the immune system plays a very important role in the gut-brain axis.

Among intestinal lymphocytes, CD4 ^+^ T cells are the main cell population mediating various host protection and homeostasis responses (82). The gut microbiota and its metabolites can directly or indirectly induce the differentiation of CD4^+^ T cells, including T-bet^+^ Th1 cells, RORγt^+^ Th17 cells, Foxp3^+^ Treg cells and GATA3^+^ Th2 cells (76). The initial polarization of CD4^+^ T cells is uncertain and dynamic, mainly characterized by the transformation of key transcription factors and characteristic cytokines. The bacteria-related inflammatory response can reprogram RORγt^+^ cells to co-express T-bet or Foxp3, promoting the interconversion between Treg cells and Th17 cells (83). The gut microbiota can influence the formation of T cell subsets through specific cytokine combinations, microbial environment, and biogeography. For example, Th13 cells that secrete IFN-γ are formed by expressing T-bet under the influence of IL-12, IL-18, and IL-23, while Th2 cells are formed under the influence of IL-4 or IL-5, express GATA3, and secrete IL-5 and IL-13 (84). Most microorganisms with epithelial cell invasion ability can be phagocytosed by dendritic cells, and can also stimulate dendritic cells to release inflammatory cytokines such as IL-6 and TNF-α, and bind with OX40 and IL-12. These signals can preferentially polarize Th1 cells (85). Th17 cells and Treg cells are two important lymphocyte subsets with opposite functions. Despite having different functional properties, differentiation from naive T cells into Th17 cells and iTreg cells is dependent on TGF-β expression levels. Low expression levels of TGF-β, IL-23, or IL-6 induced the development of naive T cells into Th17 cells, while high expression levels of TGF-β induced iTreg cells (86). The gut microbiota can induce Th17 cell response, thus inhibiting intestinal flora, while regulating Treg cell response to provide tolerance to gut microbiota (87). One study found that 11 strains of bacteria isolated from the feces of healthy people, when colonized in combination in the intestine, effectively induced IFN-γ -producing CD8^+^ T cells in the intestine and other organs of mice without causing inflammation. The induction is dependent on CD103^+^ dendritic cells and MHC Ia antigen presenting molecules (88).

## The Influence of the Gut Microbiota on the Risk Factors for Ischemic Stroke

Ischemic stroke refers to the obstruction of blood supply to brain tissue caused by various conditions, which induces irreversible damage to brain tissue at the site of insult, and leads to brain tissue necrosis (89). The risk factors for ischemic stroke are complex and diverse. The gut microbiota disorders will change the intestinal environment, affect intestinal metabolism and absorption functions, and cause gut microbiota to affect the risk factors for ischemic stroke by different means.

### Gut Microbiota and Hypertension

Hypertension has an obvious induction effect on ischemic stroke, and the incidence and prognosis of ischemic stroke are closely related to the severity degree and duration of hypertension. Therefore, blood pressure is positively associated with the incidence of stroke (90). Many studies have confirmed that hypertension is the most important independent risk factor for ischemic stroke (91, 92).

The gut microbiota can produce substances that affect blood pressure levels, which cannot be produced other organs. Butyric acid and propionic acid can induce vasodilation of the colonic and caudal arteries (93). Acetic acid is widely used in hemodialysis and is related to the development of hypotension and vascular dilatation (7). Yang et al. found that the abundance and diversity of the gut microbiota in hypertensive rats and humans decreased significantly, and the proportion of Firmicutes/Bacteroidetes in the hypertensive rat model increased (93). Another study of mice with spontaneous hypertension demonstrated increased in intestinal wall permeability, decreased intestinal content of tight junction protein, increased numbers of intestinal *Streptococcus* spp., and a significant decrease in the number of *Bifidobacterium* spp. (94). SFCAs, metabolites of gut microbiota, can regulate blood pressure by activating cell surface receptors, such as GPR41 (47) and olfactory receptor 78 (95).

### Gut Microbiota, Obesity, Diabetes

Sterile mice transplanted with normal intestinal microbiota have an increased risk of obesity and insulin resistance (96). The sensitivity of obese individuals to insulin increased significantly after receiving the gut microbiota from healthy people (97). The ratio of Firmicutes/Bacteroidetes in the intestinal tract of obese people and mice was significantly increased compared with normal people (98, 99). SFCAs are absorbed into the blood by intestinal epithelial cells and then metabolized by the liver, these metabolites can serve as a new energy source and increase energy intake and the risk of obesity (100, 101).

Hyperglycemia is an another independent risk factor for ischemic stroke, and the risk of ischemic stroke in diabetic patients is 1.8-6 times higher than in the general population (102). Changes in gut microbiota composition are related to insulin resistance. An imbalance in the gut microbiota can lead to lower insulin levels, insulin resistance, and a subsequent rise in blood glucose levels (8). Qin et al. found that there were significant differences in gut microbiota enrichment between healthy people and patients with type 2 diabetes mellitus (103). Chronic inflammation is an important pathogenic characteristic of insulin resistance. Gut microbiota imbalance may trigger an inflammatory response, and inflammatory factors may cause insulin resistance by affecting the insulin signaling pathway (104). Lipopolysaccharide can increase the production of pro-inflammatory cytokines after entering the circulation (105). These inflammatory cytokines induce the serine phosphorylation of insulin receptor substrate 1 in muscle and adipose tissue, block the insulin signaling pathway, and cause insulin resistance (106). Some anaerobic bacteria in the intestine convert primary bile acids into secondary bile acids, and a small amount of secondary bile acids can regulate lipid and glucose metabolism in the liver or the whole body by activating G-protein coupled receptors (107).

### Gut Microbiota and Atherosclerosis

Intracranial or cervical atherosclerosis is also an important risk factor for ischemic stroke, while hyperlipidemia is a risk factor for atherosclerotic plaques development. High-fat diets, hyperlipidemia, and the gut microbiota are closely related. A long-term high-fat diet may change the types of gut microbes. Relevant studies have found that hyperlipidemia can affect the metabolic status and reproduction of gut microbes, resulting in a decrease in the abundance of beneficial bacteria, such as *Bifidobacteria* and *Lactobacillus* spp., while the gut microbiota imbalance can aggravate the lipid metabolic disorders and increase blood lipid levels (108, 109). Compared with a healthy control group matched by age and gender, patients with symptomatic atherosclerosis displayed significantly more *Collinsella* sp. in the intestinal tract; the intestinal tract was rich in *Roseburia* and *Eubacterium* spp. in the healthy control group. Analysis of the functional omics of microorganisms demonstrated that the gut microbiota of patients with symptomatic atherosclerosis were rich in genes encoding the synthesis of peptidoglycan, which is the main component of the bacterial cell wall and may cause atherosclerosis by activating the immune system, particularly neutrophils (110). One study found that the bacterial DNA content in atherosclerotic plaques was related to cardiovascular risk factors, such as low-density lipoprotein cholesterol in serum (111). After transplantation of feces from atherosclerosis-sensitive mice into the intestinal tract of atherosclerosis-resistant mice, atherosclerosis occurred in the atherosclerotic resistant mice, and their serum TMAO levels increased, indicating that atherosclerotic susceptibility can be transferred through fecal microbiota transplantation (112).

## How Does Gut Microbiota Affect Ischemic Stroke Reciprocally?

Hypertension, obesity, diabetes, and atherosclerosis are independent risk factors for stroke. Gut microbiota disorders and their endotoxins and metabolites play an important role in the pathophysiological processes of obesity, diabetes mellitus, and atherosclerosis (113). In addition, increasing numbers of studies have shown that gut microbiota disorders are directly related to stroke directly.

The gut microbiota can effectively regulate the number of lymphocytes, such as regulatory T (Treg) and γδT cells, and a gut microbiota imbalance will affect Treg cells and IL-17 ^+^ γδT cells, both of which are involved in an ischemic brain injury (114). The γδT cells are a group of cells with primary innate immune function, and are situated mainly on the surface of the intestinal epithelium (115). The γδT cells can secrete large amounts of IL-17 after an imbalance in the gut microbiota, which leads to chemokine production from surrounding medullary cells (monocytes and neutrophils) and aggravation of ischemic brain injury (116). Effector T cells may induce focal ischemic brain injury, but Treg cells can play a neuroprotective role by inhibiting post-ischemic inflammation (117). Treg cells inhibit IL-17 ^+^ γδT cells by secreting IL-10, thus exerting a neuroprotective effect (118). However, Treg cells have been not shown to enter the brain parenchyma during the acute stage of stroke, indicating that the beneficial role of Treg cells is achieved by regulating the peripheral immune system rather than directly acting on damaged brain tissue (119).

Wang et al. (120) found that oral butyrate reduced brain damage caused by type 2 diabetes. Xu et al. (121) found that cerebral ischemia rapidly caused intestinal ischemia and increased production of nitrate through free radical reactions, which resulted in an intestinal flora disorder accompanied by expansion of bacteria from the family *Enterobacteriaceae*. Enrichment of *Enterobacteriaceae* microbes contributes to cerebral infarction injury by enhancing systemic inflammation and represents an independent risk factor for a major adverse outcome in stroke patients. Treatment with superoxide dismutase or aminoguanidine can reduce nitrate production, while treatment with tungstate inhibits nitrate respiration, inhibits overgrowth of *Enterobacteriaceae*, reduces systemic inflammation, and alleviates cerebral infarction injury.

The beneficial or harmful effect of gut microbiota imbalance on stroke prognosis remains unclear. Pretreatment with antibiotics inhibited the gut microbiota and significantly decreased the α-diversity of the gut microbiota by day 3 after treatment in mice with middle cerebral artery occlusion, resulting in decreased cerebral infarction volume and better prognosis (122). However, another experiment had the opposite result. In the middle cerebral artery occlusion model using C57BL/6 mice, after gut microbiota was inhibited by broad-spectrum antibiotics, the mortality rate of mice with gut microbiota disorders was significantly increased (123). Singh et al. studied the relationship between brain injury, intestinal microbial disorders, and the immune system after ischemic stroke and found that ischemic stroke caused intestinal microbial disorders and impaired gut microbiota function (1). Conversely, changes in the intestinal microbiota affected the prognosis of brain injury caused by inflammation. After ischemic stroke, T cells migrated from the intestinal tract to the damaged brain, suggesting that gut microbiota disorders may be a therapeutic target to alleviate the immune responses after ischemic stroke. Subsequently, Singh et al. used the method of intestinal bacterial transplantation to restore the balance of the gut microbiota, improve the area of cerebral infarction in mice, and reduce the number of Treg cells (1). In a study comparing the effects of oral and intravenous antibiotics on the gut microbiota, both oral and intravenous antibiotics affected the gut microbiota, taking less time to recover in the intravenous antibiotics group than in the oral antibiotics group (124).

The relationship between ischemic stroke and the gut microbiota has been extensively studied in animal models, and progress has also been made in clinical studies. Karlsson et al. studied the genomic composition of the gut microbiota in patients with cerebral ischemic stroke and found that the microbial composition was different from that of normal people. The number of *Ruminococcus* spp. was significantly increased in patients with cerebral ischemia, while the number of *Eubacillus* spp. and *Bacteroides* spp. was significantly decreased (110). The relationship between gut microbiota disorders and ischemic stroke has not been fully clarified. According to current research, gut microbiota disorders may promote the pathological disease processes and the formation of atherosclerotic plaques. A clinical study compared the gut microbiota and plasma TMAO levels in patients with asymptomatic atherosclerosis, transient ischemic stroke, and stroke. The researchers found that plasma TMAO levels and microflora composition were independent of carotid atherosclerotic plaque formation in the asymptomatic atherosclerosis group. However, the differences in microflora composition and plasma TMAO content between the transient ischemic attack and stroke groups were statistically significant (125). A study shows that patients with acute ischemic stroke can experience significant gut microbiota disturbance that lasts for more than 3 weeks, after 4 weeks, the disturbed gut microbiota can gradually recover with a significant decrease in microbiota diversity (3). The alterations in the gut microbiota may be an indication of ischemic stroke incidence, progression, and prognosis (**Table 1**).

**Table 1:** Changes in the gut microbiota composition after ischemic stroke.

Animal model or clinical study	Dysbiosis of Microbiota after ischemic stroke	Main findings	References
Clinical cohorts and mouse MCAO	Brain ischemic stroke in patients induced intestinal ischemic and produced superfluous nitrate resulting in gut disorders with *Enterobacteriaceae* extraordinary growth	Ischemic stroke induces gut dysbiosis with *Enterobacteriaceae* overgrowth that in reverse exacerbates cerebral infarction	Xu et al. (121)
Clinical cohorts	Decrease of SCFA-producing bacteria (*Roseburia*, *Lachnospiraceae*, *Bacteroides*, *Faecalibacterium*, *Blautia*, and *Anaerostipes*) and increase in opportunistic pathogens (*Porphyromonadaceae* and *Enterobacteriaceae*) and *Lactobacillaceae* and *Akkermansia* in stroke patients	Dysbiosis of SCFAs-producing bacteria and SCFAs in AIS patients raised the later risk for poor functional outcomes	Tan et al. (126)
Clinical cohorts	The gut microbiota of CI patients had more SCFAs producer including *Akkermansia*, *Odoribacter*, *Ruminococcaceae_UCG_005* and *Victivallis*.	CI patients showed significant dysbiosis of the gut microbiota with increased SCFAs producer, including *Odoribacter*, *Akkermansia*. The disorders were correlation with the LDL, HDL, blood glucose and NIHSS1M	Li et al. (127)
Clinical cohorts and mouse MCAO	In stroke patients, the proportion of *Butyricimonas*, Parabacteroides, *Rikenellaceae*, *Ruminococcaceae*, *Oscillospira*, *Bilophila*, *Enterobacteriaceae* enriched	The index of microbiota dysbiosis in ischemic stroke patients was significantly associated with patients’ outcome even prognosis and was causally related to outcome in mouse model	Xia et al. (3)
Mouse MCAO	The major characteristics of the ischemic stroke-induced in mucosal microbiota composition were enriched abundance of *Akkermansia muciniphila* and massive abundance of clostridial species.	Ischemic stroke induces significant impacts to the intestinal mucosal microbiota.	Stanley et al. (128)
Clinical cohorts	The counts of *Lactobacillus ruminis*, *Atopobium* increased and the counts of *Lactobacillus sakei* subgroup decreased.	Gut dysbiosis in AIS patients is related to host metabolism and inflammation status.	Yamashiro, et al. (129)
Mouse MCAO	Effects of cerebral injury on microbiota composition included decrease in microbiota species diversity and intestinal bacterial expansion with a preferential overgrowth of the Bacteroidetes phylum and reduction of the Firmicutes and Actinobacteria.	AIS induced dysbiosis of the microbiota and, in reverse, changes in the gut microbiota influenced neuroinflammation and functional outcomes after cerebral injury. The gut microbiota influence on immunity and ischemic stroke outcome was improvable by FMT.	Singh et al. (1)
Mouse and TBI MCAO	Cerebral injury with stroke changed the composition of *Peptococcaceae* and *Prevotellaceae* in the caecal microbiota.	Cerebral injury induced specific variations in the caecal microbiota of mice by changed mice autonomic activity and mucoprotein production.	Houlden et al. (130)
Clinical cohorts	CI and TIA patients had more opportunistic pathogens, such as *Enterobacter*, *Oscillibacter*, *Megasphaera*, and *Desulfovibrio*, also fewer commensal or beneficial genera including *Faecalibacterium*, *Prevotella*, and *Bacteroides.*	Patients with asymptomatic atherosclerosis have no clear distinctions in gut microbiota and blood TMAO levels; however, the patients with apparent dysbiosis of the gut microbiota, the TMAO concentrations in the blood were decreased.	Yin et al. (125)
Clinical cohorts	The genus *Collinsella* was enriched in patients with symptomatic carotid plaques, whereas *Eubacterium* and *Roseburia* were enriched in controls.	The gut metagenome is relevant to the host inflammation condition.	Karlsson et al. (110)

AIS, acute ischemic stroke; CI, cerebral ischemic stroke; FMT, fecal microbiota transplantation; MCAO, middle cerebral artery occlusion; SCFA, short-chain fatty acid oxide; TBI, traumatic brain injury; TIA, transient ischemic attack; TMAO, trimethylamine N-oxide.

Gut microbiota disorders and intestinal dysfunction can affect the prognosis after a stroke through various mechanisms that include microflora migration, intestinal bacterial metabolites, and immune regulation. After an ischemic stroke, intestinal motility decreases and intestinal permeability increases, and gut microbe translocation into extraintestinal organs leads to local and systemic infection (2). Endotoxins derived from the gut microbiota, such as lipopolysaccharide and peptidoglycan, enter the blood through the highly permeable intestinal wall, activate the innate immune response of the body, and aggravate the inflammatory reaction. Infection and inflammation are detrimental to stroke outcomes. After cerebral apoplexy, the host immune system is severely suppressed (131), and the immune barrier function of the intestinal tract is reduced (132). Intestinal bacteria may be transferred into the blood and external organs, thereby, inducing a systemic inflammatory response and aggravating the adverse outcome of cerebral infarction injury. Crapser et al. found that, in aged mice, ischemic stroke induced gut permeability and enhanced bacterial translocation that resulted in sepsis, while young mice were able to resolve the infection (133).

## The Possible Ways to Prevent Ischemic Stroke Through Early Intervention of Gut Microbiota

### Fecal Microbiota Transplantation for Treatment of Ischemic Stroke

Fecal microbiota transplantation (FMT) is the transplantation of functional bacteria from healthy donor feces into the gastrointestinal tract of a patient to repair the balance of the gut microbiota, a process that involves a point in time prior to the onset of disease. Filtered feces are collected from healthy donors or recipients themselves (autologous FMT) and transplanted into the intestines of patients with certain diseases (134). Chen et al. showed that transplantation of gut microbiota from normal mice into the intestinal tract of mice with ischemic stroke improved the long-term functional prognosis and survival rate (135). The results of studies on patients with severe stroke showed that the intestinal flora changed significantly after stroke onset leading to homeostasis disorders and immune responses, and FMT significantly improved the prognosis of these stroke patients (1). The mechanisms by which FMT improves the prognosis of ischemic stroke remain unclear, but is thought to be because of the restoration of intestinal homeostasis, which alleviates the inflammatory response and improves the overall condition.

FMT is still in its infancy, and it remains unknown whether FMT may result in the transfer of harmful microbes to potential subjects. There is a great deal of uncertainty regarding intestinal microbiota composition, which is complex combined with probiotics. As with any treatment, there are potential adverse effects or serious risks associated with FMT. Generally speaking, FMT is relatively safe even for individuals with low immune function or at high risk for inflammatory bowel disease. Common adverse events in the digestive tract, such as abdominal discomfort, flatulence, short-term low fever, change in defecation habits, abdominal bowel sounds, nausea, and vomiting, are mostly mild and self-limited (136). Serious adverse events, including death, are rare and are mainly caused by comorbidities or FMT manipulation problems (137). More research is needed to understand the potential side effects of FMT, such as the initiation of chronic disease or transmission of pathogens because of changes in the gut microbiota (138). FMT therapy for ischemic strokes provides a new approach, but further research will be required for extensive clinical applications.

### Probiotics and Diet for Treatment of Ischemic Stroke

Probiotics refer to edible microorganisms that are generally believed to have positive benefits to the host after ingestion, mainly including *Lactobacillus* spp. and *Bifidobacterium* spp (139). Probiotics entering the intestine from food sources will lead to the increase of SCFAs producing bacteria, resulting in a decrease in the number of protein-producing bacteria, thus promoting the increase of SCFAs and butyrate synthesis, reducing the amount of protein synthesis, resulting in changes in carbohydrate metabolism, and ultimately affecting blood glucose and blood lipids (140, 141). Probiotics such as *Lactobacillus* spp. and *Bifidobacterium* spp. can regulate renin-angiotensin system by producing angiotensin converting enzyme inhibitory peptide, SCFAs, conjugated linoleic acid and γ -aminobutyric acid, and have a certain antihypertensive effect (142). Another study showed that probiotics can treat gastrointestinal symptoms such as diarrhea and constipation after stroke (143).

Dietary phosphatidylcholine is closely related to TMAO production, and TMAO production depends on intestinal microbiota metabolism (144), while TMAO plays an important role in atherosclerosis (145). By ingesting probiotics and adjusting our daily diet, we can prevent and treat high-risk factors for ischemic stroke.

## Discussion

**Figure 1:**
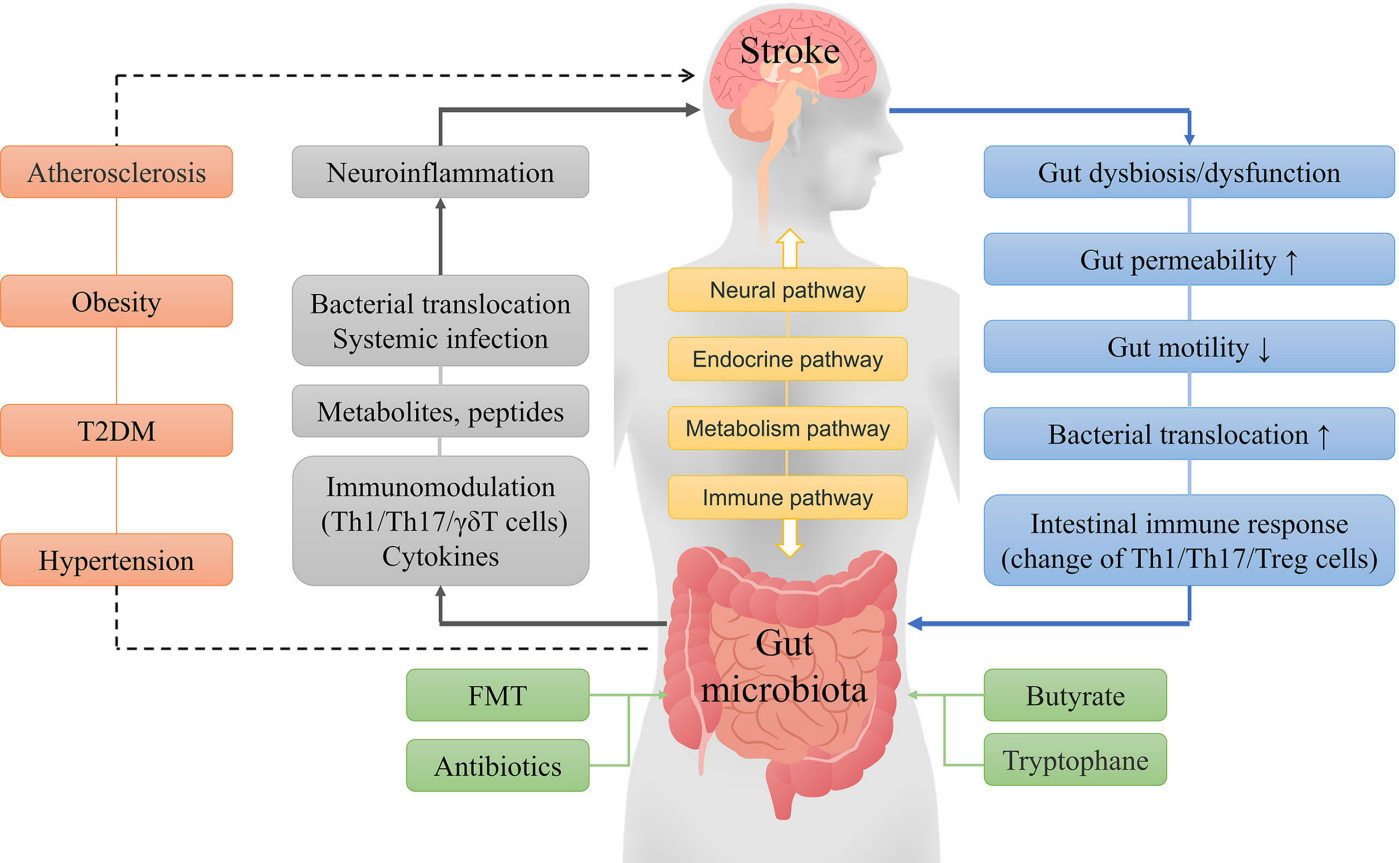
General concept of interactions between the gut microbiota and ischemic stroke. The brain and gut microbiota interact with each other through neural, endocrine, metabolic and immune pathways. A stroke can cause a series of reactions, such as gut microbiota disorder, microbial composition changes, and immune responses, while gut dysbiosis can lead to metabolic and immune response changes, systemic inflammation, and other reactions that result in increased neuroinflammation and poor stroke outcomes. Furthermore, hypertension, obesity, diabetes, and atherosclerosis are independent risk factors for stroke, and the gut microbiota may indirectly influence stroke by affecting these risk factors. Fecal microbiota transplantation, probiotics, butyrate, and tryptophane can improve the prognosis of stroke by improving the composition of the gut microbiota.

The gut microbiota can interact with the brain through various mechanisms, and gut microbiota imbalance can promote the occurrence of strokes, which in turn may aggravate the gut microbiota dysbiosis. The prognosis after a stroke can be improved through multiple interventions (**Figure 1**). The large number of gut microbes and variety of metabolites may change the impact of the gut microbiota on stroke outcomes. At present, the treatment of stroke by altering gut microbiota has substantial limitations, and more in-depth research will still be required.

There are many symbiotic microorganisms in the gut whose composition and abundance may affect both the autonomic nervous system and CNS. In recent years, many studies have confirmed that intestinal microbiome disorders are associated with certain central nervous system diseases, such as stroke, Alzheimer’s disease, Parkinson’s disease, and multiple sclerosis (146, 147). A stroke is the consequence of many complex factors, including obesity, hypertension, hyperlipidemia, insulin resistance, and atherosclerosis. Gut microbiota disorders are not only involved in the pathological processes of these risk factors, but are also directly related to strokes. A diet high in fat and sugar leads to changes in the proportion of symbiotic bacteria and imbalance of the gut microbiota (148). Therefore, maintaining a reasonable diet may reduce the incidence of ischemic stroke. After the occurrence of an ischemic stroke, the application of antibiotics for gut microbiota treatment and the restoration of intestinal homeostasis may control the development of the disease and improve its prognosis.

Although many studies have been conducted to evaluate the relationship between the gut microbiota and the occurrence and development of strokes, the following limitations remain: (1) Many studies have shown that different bacterial populations are associated with particular clinical symptoms; however, in most cases, it is not clear whether these differences cause disease. (2) Few breakthroughs have been made. Most studies have focused only on the correlations between the gut microbiota and diseases. Further studies are still needed to determine the mechanisms by which intestinal microbiota dysfunction affects the brain. (3) There is no research on chemical drugs that can effectively regulate the gut microbiota. Furthermore, the causal relationships between specific intestinal flora and specific diseases as well as detailed mechanisms should be further clarified to provide theoretical support for clinical disease prevention or treatments related to gut microbiota regulation.

The drug research on microbial flora has been performed for a considerable length of time and the present study mainly includes the following four types: (1) the FMT, which involves extracting gut microbes from healthy donor stools through different preparation processes, such as adding a protective agent to intestinal soluble capsules; (2) bacteria formula, which involves isolating strains one by one through artificial intelligence calculations and functional screenings and creating combinations of strains to treat diseases; (3) bacterial metabolites, small molecule regulators, and drugs similar to small molecules and peptides; and (4) artificial modification of bacteria through synthetic biology and genetic engineering methods to enhance the pharmaceutical function. These disease-specific microbiological drug studies require a deep understanding of the gut microbiota and the pathogenesis of various diseases, including stroke.

Microbe-targeted interventions, such as antibiotics, probiotics, and FMT, have been shown to have a beneficial effect on host health. External interventions with intestinal flora may change the outcome of some refractory diseases, including stroke, indicating that the gut microbiota has substantial therapeutic potential in clinical practice. Restoring gut microbiota homeostasis to treat ischemic stroke may represent a major breakthrough.

Future research will include defining mechanisms of microbe-microbe and microbe-host interactions in complex microbial-human gut ecosystems, using high-throughput sequencing of gut microbial genomes, and developing drugs based on these data. All of these studies will have a significant impact on treatment potential for various diseases, including stroke. Finally, it will be necessary to perform tightly designed, prospective, and longitudinal studies to transform these studies to clinical applications.

## Author Contributions

JW, HZ, and XX edited the manuscript. JH directed the project, and revised the manuscript. All authors read and approved the final manuscript.

## Funding

This work was supported by the National Natural Science Foundation of China (no. 82171336, 81870939 to XX) and Health Commission of Jiangxi Provincial (no.202130032 to JH).

## Conflict of Interest

The authors declare that the research was conducted in the absence of any commercial or financial relationships that could be construed as a potential conflict of interest.

## Publisher’s Note

All claims expressed in this article are solely those of the authors and do not necessarily represent those of their affiliated organizations, or those of the publisher, the editors and the reviewers. Any product that may be evaluated in this article, or claim that may be made by its manufacturer, is not guaranteed or endorsed by the publisher.
